# Supplementary material for: Lymphopenia in hospitalized patients and its relationship with severity of illness and mortality
Source: PLoS One. 2021 Aug 13;16(8):e0256205. doi: 10.1371/journal.pone.0256205 (PMC8362940; doi:10.1371/journal.pone.0256205)
Supplement: S1 Table — (DOCX) [file pone.0256205.s001.docx]

| **S1 Table. Significant association lymphopenia according to Diagnostic (CIE-10) (*n*=58,260)** | | | |  |
| --- | --- | --- | --- | --- |
| **Diagnostic (CIE-10)** | **Cases** | **Lymphopenia**  ***n*=23,892 (41.0%)** | **OR (CI 95%)** | **Sig.** |
|  | ***n*** | ***n* (%)** |  |  |
| **Certain_infectious_and_parasitic_diseases (A00-B99)** | **7,495** | **4,112 (54.9)** | **1.90 (1.81 – 2.00)** | **< 0.001** |
| **Neoplasm (C00-C97, D00-D09, D37-D48)** | **9,163** | **4,939 (53.9)** | **1.86 (1.78 – 1.95)** | **< 0.001** |
| -Malignant neoplasms of lip, oral cavity and pharynx (C00-C14) | 142 | 96 (67.6) | 3.01 (2.18 – 4.28) | < 0.001 |
| -Malignant neoplasms of digestive organs (C15-C26) | 2,679 | 1,431 (53.4) | 1.69 (1.56 – 1.83) | < 0.001 |
| -Malignant neoplasms of respiratory and intrathoracic organs (C30-C39) | 1,273 | 746 (58.6) | 2.07 (1.85 – 2.32) | < 0.001 |
| -Melanoma and other malignant neoplasms of skin (C43-C44) | 107 | 54 (50.5) | 1.47 (1.00 – 2.14) | 0.049 |
| -Malignant neoplasms of mesothelial and soft tissue (C45-C49) | 107 | 61 (57.0) | 1.91 (1.30 – 2.80) | 0.001 |
| -Malignant neoplasm of breast (C50) | 381 | 184 (48.3) | 1.35 (1.10 – 1.67) | 0.004 |
| -Malignant neoplasms of male genital organs (C60-C63) | 677 | 346 (51.1) | 1.51 (1.30 – 1.76) | < 0.001 |
| -Malignant neoplasms of urinary tract (C64-C68) | 1,029 | 513 (49.9) | 1.44 (1.27 – 1.63) | < 0.001 |
| -Malignant Neoplasms of ill-defined, secondary and unspecified sites (C76-C80) | 2,671 | 1,566 (58.6) | 2.11 (1.95 – 2.86) | < 0.001 |
| -Malignant Neoplasms of lymphoid haematopoietic and related tissue (C81-C96) | 1,467 | 911 (62.1) | 2.41 (2.17 – 2.68) | < 0.001 |
| -Neoplasm of uncertain or unknown behaviour (D37-D48) | 901 | 478 (53.1) | 1.64 (1.44 – 1.87) | < 0.001 |
| **Diseases of the genitourinary system (N00-N99)** | **17,586** | **8,828 (50.2)** | **1.71 (1.65 – 1.78)** | **< 0.001** |
| -Renal tubulo-intersticial diseases (N10-N16) | 736 | 362 (47.4) | 1.30 (1.13 – 1.50) | < 0.001 |
| -Renal failure (N17-N19) | 7,677 | 4,424 (57.6) | 2.17 (2.07 – 2.28) | < 0.001 |
| -Others disorders of kindey and ureter (N25-N29) | 875 | 474 (54.2) | 1.72 (1.50 – 1.96) | < 0.001 |
| -Other diseases of urinary system (N30-N39) | 5,850 | 3,025 (51.7) | 1.62 (1.53 – 1.71) | < 0.001 |
| -Diseases of male genital organs (N40-N51) | 4,589 | 2,285 (49.8) | 1.47 (1.39 – 1.56) | < 0.001 |
| **Diseases of the musculosketetal system and connective tissue** | **8,269** | **3,758 (45.4)** | **1.24 (1.18 – 1.30)** | **< 0.001** |
| -Arthropathies (M00-M25) | 5,001 | 2,378 (47.6) | 1.34 (1.26 – 1.42) | < 0.001 |
| - Systemic connective tissue disorders (M30-M36) | 787 | 366 (46.5) | 1.25 (1.09 – 1.44) | 0.002 |
| - Osteopathies and condropathies (M80-M94) | 2,092 | 932 (44.6) | 1.16 (1.06 – 1.27) | 0.001 |
| **Disease of the blood (D50-D89)** | **8,988** | **4,931 (54.9)** | **1.94 (1.86 – 2.03)** | **< 0.001** |
| -Nutritional anaemias (D50-D53) | 3,320 | 1,678 (50.5) | 1.51 (1.40 – 1.62) | < 0.001 |
| -Aplastic and other anaemias (D60-D64) | 3,881 | 2,268 (58.4) | 2.13 (1.99 – 2.78) | < 0.001 |
| - Coagulation defects, purpura and other haemorrhagic conditions (D65-D69) | 1,036 | 583 (56.3) | 1.87 (1.65 – 2.12) | < 0.001 |
| - Other diseases of blood and blood-forming organs (D70-D77) | 868 | 609 (70.2) | 3.45 (2.98 – 3.99) | < 0.001 |
| -Certain disorders involving the immune mechanism (D80-D89) | 236 | 114 (48.3) | 1.35 (1.04 – 1.74) | 0.024 |
| **Diseases of respiratory system (J00-J99)** | **18,428** | **9,830 (53.3)** | **2.10 (2.02 – 2.17)** | **< 0.001** |
| - Influenza and pneumonia (J09-J18) | 4,629 | 2,940 (63.5) | 2.72 (2.55 – 2.89) | < 0.001 |
| - Other acute lower respiratory infectious (J20-J22) | 2,924 | 1,663 (56.9) | 1.96 (1.82 – 2.12) | < 0.001 |
| - Chronic lower respiratory diseases (J40-J47) | 9,165 | 4,564 (49.8) | 1.53 (1.46 – 1.60) | < 0.001 |
| - Lung diseases due to external agents (J60-J70) | 416 | 265 (63.7) | 2.54 (2.08 – 3.11) | < 0.001 |
| - Other respiratory diseases principally affecting the interstitium (J80-J84) | 747 | 423 (56.6) | 1.89 (1.64 – 2.19) | < 0.001 |
| - Other diseases of pleura (J90-J94) | 1,357 | 767 (56.5) | 1.90(1.70 – 2.12) | < 0.001 |
| - Other diseases of respiratory system (J95-J99) | 6,498 | 3,902 (60.0) | 2.39 (2.27 – 2.52) | < 0.001 |
| **Diseases of digestive system (K00-K87).** | **16,129** | **7,330 (45.4)** | **1.29 (1.24 – 1.34)** | **< 0.001** |
| - Diseases of oral cavity, salivary glands and jaws (K00-K14) | 252 | 135 (53.6) | 1.66 (1.30 – 2.13) | < 0.001 |
| - Diseases of oesophagus, stomach and duodenum (K20-K31) | 2,839 | 1232 (43.4) | 1.11 (1.03 – 1.20) | 0.008 |
| - Hernia (K40-K46) | 2,950 | 1,329 (45.1) | 1.19 (1.11 – 1.28) | < 0.001 |
| - Noninfective enteritis and colitis (K50-K52) | 1,370 | 618 (45.1) | 1.19 (1.07 – 1.32) | 0.002 |
| - Others diseases of intestines (K55-K64) | 5,013 | 2,219 (44.3) | 1.16 (1.09 – 1.23) | < 0.001 |
| - Diseases of peritoneum (K65-K67) | 552 | 344 (62.3) | 2.40 (2.02 – 2.85) | < 0.001 |
| - Diseases of liver (K70-K77) | 2,917 | 1,536 (52.7) | 1.64 (1.52 – 1.77) | < 0.001 |
| - Disorders of gallbladder, biliary tract and pancreas (K80-K87) | 3,543 | 1,823 (51.5) | 1.57 (1.47 – 1.68) | < 0.001 |
