# Supplementary material for: Lymphopenia in hospitalized patients and its relationship with severity of illness and mortality
Source: PLoS One. 2021 Aug 13;16(8):e0256205. doi: 10.1371/journal.pone.0256205 (PMC8362940; doi:10.1371/journal.pone.0256205)
Supplement: S2 Table — (DOCX) [file pone.0256205.s002.docx]

| **S2 Table. Significant association lymphopenia according to Infectious Diseases (CIE-10).** | | | | |
| --- | --- | --- | --- | --- |
|  | **Cases** | **Lymphopenia** | **OR (CI 95%)** | **Sig** |
|  | ***n*** | ***n* (%)** |  |  |
| **Infectious Diseases** | **21,093** | **11,016 (52.2)** | **2.06 (1.99 – 2.14)** | **< 0.001** |
| **Certain infectious and parasitic diseases** (A00-B99) | **7,495** | **4,112 (54.9)** | **1.90 (1.81 – 2.00)** | **< 0.001** |
| --Intestinal infectious diseases (A00-09) | 323 | 158 (48.9) | 1.38 (1.11 – 1.18) | 0.004 |
| --Other bacterial diseases (A30-49) | 1,274 | 996 (78.2) | 5.33 (4.67 – 6.10) | < 0.001 |
| --Viral infections, skin and mucous membrane lesions (B00-09) | 243 | 125 (51.4) | 1.53 (1.19 – 1.96) | 0.001 |
| --Viral hepatitis (B15-19) | 1,325 | 590 (44.5) | 1.16 (1.04 – 1.29) | 0.008 |
| --Mycoses (B35-49) | 605 | 337 (55.7) | 1.82 (1.55 – 2.14) | < 0.001 |
| --Protozoal Disease (B50-64) | 85 | 52 (61.2) | 2.27 (1.47 – 3.51) | < 0.001 |
| --Sequelae of infectious and parasitic diseases (B90-94) | 315 | 166 (52.7) | 1.61 (1.29 – 2.01) | < 0.001 |
| --Bacterial, viral and other infectious agents (B95-98) | 3,363 | 1,923 (57.2) | 2.00 (1.87 – 2.15) | < 0.001 |
| **Respiratory Infectious diseases** | **8,221** | **4,777 (58.1)** | **2.24 (2.14 – 2.35)** | **< 0.001** |
| --Influenza and pneumonia (J09-18) | 4,629 | 2,940 (63.5) | 2.72 (2.55 – 2.89) | < 0.001 |
| ---Bacterial pneumonia | 4,473 | 2824 (63.1) | 2.66 (2.50 – 2.83) | < 0.001 |
| ---Viral pneumonia | 124 | 92 (74.2) | 4.15 (2.77 – 6.20) | < 0.001 |
| --Other acute lower respiratory infectious (J20-22) | 2,924 | 1,663 (56.9) | 1.96 (1.82 – 2.12) | < 0.001 |
| **Digestive infectious diseases** | **813** | **527 (64.8)** | **2.69 (2.33 – 3.11)** | **< 0.001** |
| --Peritonitis (K65) | 252 | 171 (67.9) | 3.05 (2.34 – 3.98) | < 0.001 |
| --Cholecystitis Cholangitis (K81-83) | 577 | 369 (64.0) | 2.58 (2.17 – 3.06) | < 0.001 |
| **Genitourinary system infections** | **10,000** | **4,983 (49.8)** | **1.54 (1.48 – 1.61)** | **< 0.001** |
| --Urinary Tract Infection, site not specified (N39.0) | 5,417 | 2,869 (53.0) | 1.70 (1.61 – 1.80) | < 0.001 |
| --Diseases of male genital organs (N40-51) | 4,589 | 2,285 (49.8) | 1.47 (1.39 – 1.56) | < 0.001 |
| --Inflammatory diseases of female pelvic organs (N70-77) | 301 | 77 (25.6) | 0.49 (0.38 – 0.64) | < 0.001 |
| **Infectious diseases of central nervous system (G00-07)** | **65** | **40 (61.5)** | **2.30 (1.40 – 3.80)** | **0.001** |
| **Infectious of the skin and subcutaneous tissue** **(L00-08)** | **39** | **23 (59.0)** | **2.07 (1.09 – 3.92)** | **0.032** |
| **SEPSIS** | **6,208** | **3,560 (56.0)** | **2.06 (1.96 – 2.18)** | **< 0.001** |
| **SEPTIC SHOCK (R65.21)** | **515** | **446 (86.6)** | **9.46 (7.33 – 12.19)** | **< 0.001** |
