# Supplementary material for: Lymphopenia in hospitalized patients and its relationship with severity of illness and mortality
Source: PLoS One. 2021 Aug 13;16(8):e0256205. doi: 10.1371/journal.pone.0256205 (PMC8362940; doi:10.1371/journal.pone.0256205)
Supplement: S1 Appendix — Each patient can have multiple diagnoses, so there are more diagnoses than patients. S1 Appendix shows the number of patients who have one or more diagnoses at the same time according to disease groups. Finally, it can see that the sum of the totals is equal to the sum of the diagnoses. (DOCX) [file pone.0256205.s003.docx]

| **S1 Appendix. Number of diagnoses in each patient according to disease groups** | | |
| --- | --- | --- |
| **Diseases Groups** | **N in each patient** | **N** |
| Certain infectious and parasitic diseases | **Total** | **7,495** |
|  | 1 | 6,445 |
|  | 2 | 950 |
|  | 3 | 93 |
|  | 4 | 7 |
| Neoplasm | **Total** | **9,163** |
|  | 1 | 6,500 |
|  | 2 | 2,490 |
|  | 3 | 170 |
|  | 4 | 3 |
| Disease of blood | **Total** | **8,988** |
|  | 1 | 8,399 |
|  | 2 | 535 |
|  | 3 | 52 |
|  | 4 | 2 |
| Diseases of respiratory sistem | **Total** | **18,428** |
|  | 1 | 11,714 |
|  | 2 | 5,302 |
|  | 3 | 1,310 |
|  | 4 | 100 |
|  | 5 | 1 |
|  | 6 | 1 |
| Diseases of digestive system | **Total** | **16,129** |
|  | 1 | 12,641 |
|  | 2 | 2,911 |
|  | 3 | 516 |
|  | 4 | 61 |
| Diseases of the musculoskeletal system and connective tissue | **Total** | **8,269** |
|  | 1 | 6,634 |
|  | 2 | 1,425 |
|  | 3 | 190 |
|  | 4 | 20 |
| Diseases of the genitourinary system | **Total** | **17,586** |
|  | 1 | 14,231 |
|  | 2 | 3,000 |
|  | 3 | 341 |
|  | 4 | 13 |
|  | 5 | 1 |
| Infectious Diseases (Table 2). Except Sepsis and Septic Shock | **Total** | **21,093** |
|  | 1 | 15,937 |
|  | 2 | 4,733 |
|  | 3 | 420 |
|  | 4 | 3 |
